# Carceral Health is Public Health

**DOI:** 10.3201/eid3013.240258

**Published:** 2024-04

**Authors:** Liesl M. Hagan, Emily Mosites, Laura Hughes-Baker, Jay Butler

**Affiliations:** Centers for Disease Control and Prevention, Georgia, USA

**Keywords:** COVID-19, respiratory infections, SARS-CoV-2, SARS, coronavirus disease, zoonoses, viruses, bacteria, tuberculosis, TB, coronavirus carceral health, prison, jail, detention, incarceration

The incarceration rate in the United States is the highest in the world, 664 persons per 100,000 population in 2021 (*1*,*2*). The Bureau of Justice Statistics estimated that >5.5 million persons were under the supervision of US adult carceral systems at a single point in 2021, including persons in prison or jail and those on probation or parole (*1*,*2*). In total, ≈450,000 persons were released from state and federal prisons during 2021, and another 7 million cycled through local jails, each returning to their families and communities (*3*,*4*).

The effects of incarceration extend well beyond those who have been confined. Approximately 400,000 staff work within carceral facilities as corrections officers, and many more work as healthcare providers, administrators, educators, volunteers, and in other roles (*5*). Outside of the facilities themselves, ≈113 million adults, half of all adults in the United States, have an immediate family member who has been held in a prison or jail for at least 1 night (*6*).

Simply stated, carceral health is public health. Every person’s health has inherent value, and their wellbeing matters to them and to their loved ones, regardless of whether they are incarcerated. In addition, the walls of prisons and jails are porous, and 2-way movement of both people and pathogens affects the entire community.

The articles in this supplement of *Emerging Infectious Diseases*, Infectious Diseases and Carceral Health, explore ways that persons with infectious disease risks are concentrated within carceral systems, how the physical environment and culture within facilities can contribute to disease spread, the wide variations in access to healthcare during confinement, and implications that those compounding factors have on re-entry and broader community health. Many articles identify actionable ways to address those challenges and to promote a mainstream understanding of carceral health as a critical component of public health.

The first several articles focus on COVID-19, both to understand the pandemic’s disproportionate effects on incarcerated persons and staff working in carceral settings and to demonstrate the pressing improvements needed to address carceral public health broadly. Waddell et al. provide a national picture of successes and challenges during responses to COVID-19 in carceral facilities through qualitative interviews with staff from facilities and health departments across the country (*7*). Two additional articles provide state perspectives by focusing on specific scenarios. Tunstall et al. describe the work of a multidisciplinary governmental team in Colorado to keep youth and staff in juvenile facilities safe (*8*). Gurrey et al. describe how partnerships forged between the Washington State Department of Health and Department of Corrections during the pandemic supported their joint response to a large tuberculosis outbreak in the state’s adult prison system during 2021–2022 (*9*).

Although the COVID-19 pandemic catalyzed and invigorated carceral–public health partnerships in many jurisdictions, it also revealed the patchwork coverage of carceral systems within existing public health data sources at local, state, and national levels. Two articles in this supplement illustrate creative public health surveillance strategies that facilities and health departments developed to fill those data gaps, and potential applications to other infectious diseases. Saber et al. describe a COVID-19 wastewater testing program developed through a partnership between academic researchers and a local urban jail in Georgia (*10*), and Porter et al. evaluate a semiautomated strategy to identify COVID-19 cases associated with local jails in Minnesota by using keyword matching within case reports and electronic laboratory reports received by the health department (*11*).

In addition to COVID-19, this supplement highlights 3 infectious disease outbreaks and case summaries in state prisons and local jails as examples of responses to emerging health threats in carceral settings. Hennessee et al. present data on recent *Candida auris* cases among persons confined in state prisons in multiple states and describe considerations for infection control in healthcare and non-healthcare spaces within those facilities (*12*). Kamali et al. describe an outbreak of invasive *Serratia marcescens* in a California state prison in which genomic sequencing was used to identify contact patterns and possible transmission sources (*13*). Hassan et al. present findings from qualitative interviews with staff and persons detained in the Cook County Jail in Chicago, Illinois, after potential exposure to mpox (*14*), highlighting the need to include persons living and working in affected facilities when developing disease prevention programs and response strategies.

Several articles discuss access to everyday infectious disease prevention, another key facet of carceral health. Laryea-Adekimi et al. describe a process to develop vaccine education messaging and identify communication methods preferred by persons incarcerated in prisons in several countries in Europe (*15*). Wolf et al. document successes and challenges in implementing a routine screening program for sexually transmitted infections among youth detained in Utah juvenile facilities (*16*). Two articles focus on preexposure prophylaxis (PrEP) to prevent HIV infection, from different vantage points. Nijhawan et al. present findings from a study among persons recently released from confinement (*17*), demonstrating high prevalence of risk factors for HIV combined with low awareness of their risk. From the other side of the walls, Huang et al. describe the development and early results from a novel PrEP program available to persons incarcerated within the Federal Bureau of Prisons as part of re-entry planning (*18*). To conclude that section, McNamara et al. review the evolution of policy and practice for hepatitis C testing and treatment within prisons and jails (*19*), emphasizing the importance of carceral settings in hepatitis C elimination throughout the United States.

The final 2 articles emphasize the potential influence of facility and public policy on the future of carceral health. Kendig et al. make the case for developing comprehensive infection prevention and control programs tailored to the unique implementation challenges within carceral environments and discuss the need to invest in staff training and enhanced carceral–public health collaborations (*20*). Finally, Wurcel et al. describe recent trends in state-level efforts to improve access to healthcare during confinement and continuity of care after release through waivers of the Medicaid Inmate Exclusion Policy (*21*).

Collectively, the articles in this supplement highlight 4 pressing needs to improve carceral public health. First, partnerships among health, social service, and carceral agencies need to improve at all jurisdictional levels. Lessons from COVID-19 highlight a variety of ways to achieve this goal, and organizations found success when they broadened the definition of who their partners are or should be, not only outside their organizations but also within them, including incarcerated persons (*8*,*10*,*14*,*15*).

Second, carceral and public health partners need to share data to reach common goals, including readiness for the next public health threat (*7*–*11*). When facilities participate fully in public health surveillance, infectious disease outbreaks in prisons and jails can be identified earlier, preventing illness and death and allowing the facility to return to normal operations faster. Through sustained communication and data transparency, health departments can muster resources to support carceral facilities during public health emergencies and can offer training and assistance to prevent infectious disease outbreaks.

Third, access to preventive healthcare during incarceration needs to expand. A movement toward caring for the overall health of persons during confinement has been growing in recent years, and more carceral administrators now view the health of the populations under their care and custody as part of their responsibilities. To continue that momentum, expanding opportunities for preventive healthcare interventions during confinement and enabling continuity of care during transitions back into the community are essential.

Finally, to effectively collaborate, share data, and support healthcare in carceral settings, public health and carceral systems need to better understand one another. A growing number of health departments are making the decision to dedicate staff to public health needs within carceral facilities—positions largely funded through time-limited COVID-19 appropriations—and some carceral systems are likewise investing in maintaining staff focused on population health. Some states have also embedded health department staff within facilities, supporting those positions jointly through the department of health and department of corrections. However, those examples are not the norm. Sustained funding is needed to scale up and support such investments for the long term.

As the articles in this supplement demonstrate, carceral facilities do not exist in isolation; instead, quite the opposite is true. The health of persons living and working in those settings reverberates through communities across the country in myriad ways—when staff return home from work each day, when incarcerated persons are transferred between facilities in different jurisdictions or participate in work release programs, and when most eventually are released to reunite with their families. As a society, our varied agencies and institutions—and we as individual practitioners, staff, and community members—each have a role to play in safeguarding and improving people’s health during confinement, supporting their continued health and success after release and, ultimately, encouraging alternatives to incarceration and evolution of criminal justice systems to better support public health.
